# Role of BRCA Mutation and HE4 in Predicting Chemotherapy Response in Ovarian Cancer: A Retrospective Pilot Study

**DOI:** 10.3390/biomedicines9010055

**Published:** 2021-01-08

**Authors:** Francesco Plotti, Corrado Terranova, Federica Guzzo, Carlo De Cicco Nardone, Daniela Luvero, Martina Bartolone, Camilla Dionisi, Domenico Benvenuto, Silvia Fabris, Massimo Ciccozzi, Violante Di Donato, Pierluigi Benedetti Panici, Roberto Angioli

**Affiliations:** 1 Department of Obstetrics and Gynecology, University ‘‘Campus Bio-Medico’’ of Rome, Via Alvaro del Portillo, 200-00128 Rome, Italy; f.plotti@unicampus.it (F.P.); c.terranova@unicampus.it (C.T.); f.guzzo@unicampus.it (F.G.); c.decicconardone@unicampus.it (C.D.C.N.); d.luvero@unicampus.it (D.L.); m.bartolone@unicampus.it (M.B.); r.angioli@unicampus.it (R.A.); 2 Unit of Medical Statistics and Molecular Epidemiology, University Campus Bio-Medico of Rome, 00128 Rome, Italy; d.benvenuto@unicampus.it (D.B.); silviafabris3@yahoo.it (S.F.); m.ciccozzi@unicampus.it (M.C.); 3 Department of Gynecology, Obstetrics and Urology, Policlinico Umberto I, “Sapienza” University of Rome, 00161 Rome, Italy; violante.didonato@uniroma1.it (V.D.D.); pierluigi.benedettipanici@uniroma1.it (P.B.P.)

**Keywords:** ovarian cancer, HE4, *BRCA*, chemotherapy response

## Abstract

Even though 80% of patients with High-Grade Serous Ovarian Cancer respond to standard first-line chemotherapy, a majority of them could relapse in the following five years due to a resistance to platinum. Human Epididymis protein 4 (HE4) is one of the most promising markers in predicting platinum therapy response. This pilot study aims to evaluate the potential role of HE4 value in predicting chemotherapy response in *BRCA* mutated patients and in *BRCA* wild-type (non-mutated) ones. We selected 69 patients, affected by High-Grade Serous Ovarian Cancer, and optimally debulked and submitted to standard chemotherapy protocols. HE4 was dosed during every chemotherapy course. Patients were classified as platinum-resistant and platinum-sensitive. According to *BRCA* mutation test, patients were further divided into *BRCA* wild-type (53 patients), and *BRCA* mutated (16 patients). 35 patients out of 69 (52%) were platinum-sensitive (recurrence > 12 months), while 33 patients (48%) were platinum-resistant (recurrence < 12 months). Thus, in the total population, HE4 performed as a marker of chemosensitivity with a sensibility of 79% and a specificity of 97%. In the *BRCA* WT group, 23 patients out of 53 (43%) were platinum-sensitive, while 30 patients out of 53 (57%) were platinum-resistant. In the *BRCA* WT group, HE4 performed as a predictive marker of chemosensitivity with a sensibility of 80% and a specificity of 100%. In the *BRCA* mutated group, 13 patients out of 16 (82%) were platinum-sensitive, while 3 patients (18%) were platinum-resistant. In the *BRCA* mutated group, HE4 performed as a predictive marker of chemosensitivity in all patients. The ability to detect platinum-resistant patients before tumor relapse probably could open new therapeutic scenarios.

## 1. Introduction

Despite several advances in cytoreductive surgery and the use of first-line chemotherapy (currently consisting of carboplatin/paclitaxel), ovarian cancer is the seventh most common cancer in women and is the most lethal gynecologic malignancy, with five-year survival rates below 45% [1]. Histologically, about 90% of ovarian tumors are Epithelial Ovarian Cancers (EOCs). Among those, High-Grade Serous Ovarian Cancer (HGSOC) represents the most common type. Patients affected by HGSOC initially respond favorably to platinum-based chemotherapy, but more than 80% could relapse at some stage [2].

There is a number of known risk-factors that may modify the risk of developing ovarian cancer in individuals [3].

They include hormonal risk factors [4], modifiable, or lifestyle determinants [1], and genetic factors. The risk for women with an affected first-degree relative is threefold greater than the one of women without any affected relative [5]. Overall, it was estimated that germline *BRCA1* and *BRCA2* mutations contribute to the development of 10–20% of EOCs.

Both *BRCA1* and *BRCA2* are tumor suppressor genes, since they modulate the response to cellular stress via the activation of DNA repair processes. In particular, *BRCA1* has an effect on several cellular pathways, such as DNA damage repair, cell-cycle arrest, apoptosis, genetic instability, transcriptional activation, and tumorigenesis. *BRCA2* determines inefficient and aberrant chromosomal structures, via the maintenance of genome stability [5].

The cancer occurring in these women is usually a high-grade serous carcinoma, which manifests at an earlier age than in sporadic cases [1].

Compared to the normal population risk of about 1.4%, *BRCA1* mutation carriers have an estimated 40% risk of developing ovarian cancer by age 70, while this risk is up to 18% for *BRCA2* mutant individuals [6].

In an analysis carried out by The Cancer Genome Atlas Research Network on 316 patients affected by HGSOC, *BRCA1* and *BRCA2* germline mutations were identified in 9% and 8% of cases, respectively, and an additional 3% showed somatic mutations of the *BRCA* genes, for a total of 20% of HGS ovarian carcinomas exhibiting a *BRCA* mutation. Moreover, it is estimated that for every 4–5 ovarian cancer patients showing a germline *BRCA* mutation, there could potentially be one patient with only somatic *BRCA* mutation (without a germline mutation) [7,8].

However, even if having a *BRCA* mutation is a well-known risk factor, among individuals with OC (ovarian cancer), both *BRCA1* and *BRCA2*-associated patients have better outcomes.

A recent study demonstrated that the progression-free survival (PFS) was significantly longer in the *BRCA1* and *BRCA2* group compared to the sporadic group, namely, 2.1, 5.6, and 1.3 years, respectively [9].

In EOC, serological markers as Cancer Antigen 125 (CA125) and Human Epididymis protein 4 (HE4) play an important role in the diagnosis, in the monitoring of relapse, and in the prognosis of the disease.

Follow-up consists, as recommended by the National Comprehensive Cancer Network (NCCN) guideline, in medical examination plus CA125 and HE4 dosage every 2 to 4 months for two years, then 3 to 6 months for three years, then annually after five years. CA-125 sensitivity to detect ovarian cancer recurrence amounts to 83.9% [10], but it is negative in 50% of ovarian cancer early stages, and in 10% of advanced stages [11,12]. 

HE4, firstly identified in 1991 by Kirchoff and isolated in the epididymis, is a secretory glycoprotein, weakly expressed in the epithelium tissues of respiratory and reproductive organs, but it is overexpressed in ovarian tumors [13]. 

Literature shows that HE4 has a diagnostic sensitivity and specificity similar to CA125 in patients with gynecological malignancies, while a recent systematic review shows that HE4 has a sensitivity of 64 % (0.61–0.70) in the early diagnosis of EOC, much higher than CA125 (45.9%) [14]. 

Recently HE4 (cut-off 70 pmol/L) has been shown to be a promising marker, still under investigation, alone or in combination with CA-125, for ovarian cancers follow-up, showing 76.47% sensitivity and 100% specificity [14,15]. Furthermore, HE4 seems to be useful to successfully identify patients with poorer prognosis and recurrence in CA125 negative ovarian cancer patients [16].

Furthermore, HE4 was demonstrated to be an earlier indicator of recurrence of OC in comparison to CA-125, with a lead time of 5 to 8 months. Moreover, the sensitivity and specificity of HE4, alone or in association with other markers (CA-125, CA-72.4), seem to be higher in the diagnosis of the OC relapse in comparison to CA-125 alone, as reported by our group [14].

To date, CA125 alone showed a specificity of 78%, but resulted in be altered in patients affected by endometriosis, while HE4 levels are not influenced by the presence of endometriomas, and his specificity was 86% in an analysis by Yanaranop et al. [16].

Interestingly, several studies showed a potential role of HE4 in predicting platinum therapy response both with “in vivo clinical studies” [15,17,18,19,20] and “in vitro studies” [21,22,23,24,25], which our group deeply analyzed in a previous review [26]. However, there are no published studies about the role of HE4 during the follow-up among *BRCA* individuals.

Therefore, the aim of the present pilot study is to evaluate the potential role of HE4 value in predicting chemotherapy response in *BRCA* mutated patients and in *BRCA* wild-type ones, hoping to enroll a larger cohort of patients in the future.

## 2. Experimental Section

We conducted a retrospective study from January 2011 to June 2020, enrolling all patients referred to the Division of Gynecologic Oncology of the University Campus Bio-Medico of Rome, affected by EOC.

### 2.1. Inclusion Criteria Were

I. Age between 18 and 70 years
II. HE4 test positive at diagnosis
III. Eastern Cooperative Oncology Group (ECOG) performance status 0–2 according to World Health Organization (WHO) criteria
IV. Residual tumor after surgical debulking < 1(RT < 1)
V. No previous surgical, chemotherapeutic, or radiotherapeutic treatments for this or other kinds of cancer

### 2.2. Exclusion Criteria Were

I Altered hepatic function (transaminases > 2.5× the upper normal level –UNL-, bilirubin > 1.5× UNL)
II Altered renal function (creatinine clearance < 60 mL/min and/or serum creatinine > 2.0 mg/100 mL)
III Altered hematological function (absolute neutrophil count < 1.5 × 10^9^/L or platelets < 100 × 10^9^/L or hemoglobin < 9 g/dL)
IV Severe or uncontrolled diseases, other systemic and not compensated diseases or mental illnesses at the diagnosis
V Pregnancy

All patients recruited were tested for somatic *BRCA1/2* mutation, regardless of family history. *BRCA* analysis has been performed by Next Generation Sequencing (NGS) and interpreted using an allelic load threshold of 5%. There was no variant of uncertain significance (VUS).

All patients have been submitted to cytoreductive surgery and started the same line of chemotherapy (CT), according to standard Protocols (Carboplatin and Taxol), within 30 days from surgery [27,28,29,30]. Carboplatin doses were calculated according to the Calvert formula, with target AUC (Area Under the Curve) 6. Paclitaxel dose was 175 mg/mq.

We considered 70 pmol/L as the cut-off for HE4 serum levels.

HE4 and CA125 were dosed routinely at the diagnosis, before surgical debulking, and during every chemotherapy course. CA125 was measured using standard radioimmunoassay dosage (normal value < 35 IU/mL) during the whole study period. HE4 was measured using ELISA test (enzyme immunometric assay for the quantitative determination of HE4 in human serum), in particular the kit HE4 EIA, produced by Fujirebio Diagnostic Inc. (Malvem, PA, USA). It is a direct, not competitive, immunoblot assay, in the solid phase, based on the “sandwich” technique: It consists of the use of two murine monoclonal antibodies (2H5 and 3D8) directed against two HE4 epitopes in the C-WFDC domain.

As suggested by Gynecologic Cancer Intergroup (GCIG), the response was evaluated using RECIST 1.1 criteria. For each patient, we took into consideration HE4 values before surgery, and at each chemotherapy course; in particular, we focused on the third chemotherapy HE4 value in the disease recurrence.

Between the end of chemotherapy treatment and disease recurrence, patients were classified in:

I Platinum-resistant: Disease progression during chemotherapy treatment or within 12 months after the end of chemotherapy.
II Platinum-sensitive: Recurrence after 12 months or more.

We studied, in terms of sensibility and specificity, how a positive or negative value of HE4 at the third course of chemotherapy, can be a good predictor of platinum-sensitivity or resistance. The same analysis has been repeated based on *BRCA* mutation, dividing patients into two groups: *BRCA* mutated and *BRCA* wild-type.

To explore independence among tumoral stages and the presence or absence of *BRCA* mutation, we used the Chi-Squared test, and we assess independence with a *p* value greater than 0.05. The same test has been used to evaluate the presence of residual tumors (RT) between the two groups.

Finally, a logistic regression analysis has been performed to find clinical factors correlated with platinum-resistance and tumor relapse. The receiving operator (ROC) has been performed using HE4 levels before surgery and during every chemotherapy course, since the HE4 measurements were not available for every chemotherapy course using as a classification variable the relapse of cancer.

Using the initial database, the receiver operating characteristic curve (ROC) was performed using HE4 levels before surgery and during every cycle of chemotherapy circle; using the relapse of cancer as a classification variable, only the results regarding the measurements of HE4 before the surgery, during the third and the sixth cycle of chemotherapy were shown. Furthermore, the dataset has been divided into two databases according to the *BRCA* test, only HE4 levels before surgery and during the third cycle of chemotherapy circle had enough data to perform the ROC analysis. This analysis has been made to estimate the prognostic value of HE4 in ovarian cancer both in patients with a mutation of *BRCA* genes and in patients without a *BRCA* mutation. Sensitivity, specificity, and area under the curve (AUC) have been assessed. The HE4 variations over time for each patient have been shown using Excel software. The evolution of HE4 has been evaluated in the groups and has been compared using the *t*-student test with Welch correction test in order to find statistically significant differences among patients with *BRCA* mutation and patients without *BRCA* mutation. Statistical analysis has been performed using GraphPad Prism version 7.00 for Windows, GraphPad Software, La Jolla California USA, www.graphpad.com and Statistical Software version 19.1.3 (MedCalc Software bv, Ostend, Belgium; https://www.medcalc.org; 2019). The *p* value < 0.005 was considered statistically significant.

## 3. Results

**Figure 1:**
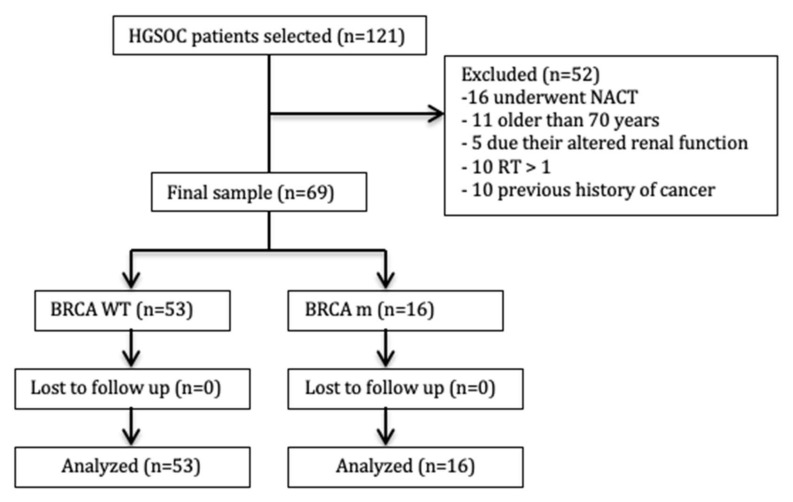
Study enrollment flowchart. *BRCA* WT = *BRCA* wild-type group’; *BRCA* m *= BRCA* mutated group.

In this retrospective pilot study, we selected 121 patients affected by EOC, all tested for somatic *BRCA1/2* mutation. According to exclusion criteria, 16 patients were excluded because they underwent neoadjuvant chemotherapy (NACT), 11 were excluded because older than 70 years, 5 were excluded based on altered renal function, 10 were excluded for Residual Tumor > 1 and 10 were excluded based on a previous cancer (Figure 1).

The final sample consisted of 69 patients, optimally debulked. Because of the small number of patients, all results reported below need to be confirmed in the future with other studies. All patients were affected by the same histological subtype, High-Grade Serous Ovarian Cancer (HGSOC). Patients’ age, Performance Status (PS), FIGO (International Federation of Gynecology and Obstetrics) stage, post-surgery RT, histological subtype, and platinum response are reported in Table 1.

### 3.1. All Population

The average preoperative HE4 value was 882.68 (70–12,630) pmol/L. The average value of HE4 at the III chemotherapy course was 198.7 (21–1217.3) pmol/L. After six courses of CT, patients underwent a follow-up, and were divided into platinum-resistant or platinum-sensitive. In particular, 36 patients out of 69 (52%) were platinum-sensitive (recurrence > 12 months), while 33 patients (48%) were platinum-resistant (recurrence < 12 months).

We chose to consider only data at pre-surgery and at the third cycle, because they are the most used values in literature, together with the value at the VI cycle. The value at the VI cycle is not significant in predicting response to platinum-based chemotherapy though (*p* = ns).

HE4 value at the III chemotherapy course was negative in 42 patients out of 69 (61%), while it remained positive in 27 patients (39%) (Table 2).

Among the patients with negative HE4 at the III CT course, 83% (35 patients) were platinum-sensitive, while 17% (7 patients) were platinum-resistant. On the contrary, among the 27 patients with positive HE4 at the III CT course, 97% (26 patients) were platinum-resistant. Thus, in all the population, HE4 performed as a predictive marker of chemosensitivity with a sensibility of 79% and a specificity of 97%.

According to *BRCA* mutation test, patients were further divided into two groups: *BRCA* wild-type (53 patients) and *BRCA* mutated (16 patients). 

### 3.2. BRCA WT Group

In *BRCA* WT the average preoperative HE4 value was 843.2 (81.3–126,30) pmol/L, and the average value of HE4 at the III chemotherapy course was 233 (21.3–4096) pmol/L. In the *BRCA* WT group, 23 patients out of 53 (43%) were platinum-sensitive, while 30 patients out of 53 (57%) were platinum-resistant (Table 3).

Considering the HE4 value at the III chemotherapy course, 29 out of 53 patients (55%) exhibited a negative value, while 24 out of 53 patients (45%) exhibited a positive value. Among the 29 patients with a negative HE4 at the III course, 22 patients (76%) experienced recurrence after 12 months, and 7 patients (24%) within 0–12 months. Among the 24 patients with a positive HE4, on the contrary, 23 patients (96%) experienced a recurrence within 12 months, and 1 patient (4%) after 12 months.

In the *BRCA* WT group, HE4 performed as a predictive marker of chemosensitivity with a sensibility of 80% and a specificity of 100%.

### 3.3. BRCA Mutated Group

In *BRCA* mutated group, the average preoperative HE4 value was 293.8 (71–1498) pmol/L, and the average value of HE4 at the III chemotherapy course was 52.81 (30.1–87.8) pmol/L.

In *BRCA* mutated group, 13 patients out of 16 (82%) were platinum-sensitive, while 3 patients (18%) were platinum-resistant (Table 4).

Considering HE4 value at the III chemotherapy course, in *BRCA* mutated patients, 13 out of 16 patients (81%) exhibited a negative value, 3 out of 16 patients (19%) exhibited a positive value. All patients with a negative HE4 value at the III course were platinum-sensitive. Any patient with a negative HE4 value at the III course was platinum-resistant. The three patients that exhibited a positive value of HE4 at the III course were all platinum-resistant.

In the *BRCA* mutated group, HE4 performed as a predictive marker of chemosensitivity with a sensibility of 100% and a specificity of 100%.

Then we performed logistic regression analysis of factor predicting recurrences before 12 months and resulted that He4 > 70 pmol/L after the third cycle of CT is correlated with Recurrence > 12 months (odds ratio = 28; 95% CI (Confidence interval= 4.55–183.18; *p* value < 0.0001).

Moreover, in all populations, the statistical analysis suggests that the best balance between sensitivity and specificity is the level of HE4 after the 3rd cycle.

In fact, in *BRCA* mutated population, a level of HE4 higher than 65,6pmol/L after the third cycle of chemotherapy seems to be an effective prognostic marker to identify those patients who will develop an ovarian cancer relapse (sensitivity = 75; specificity= 99; AUC = 92%, *p* value < 0.001; LR+ = 750; Post-test probability = 99%) (Table 5).

**Figure 2:**
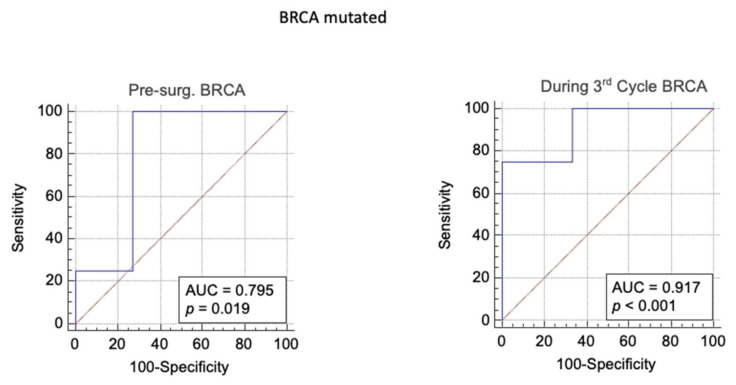
*BRCA* population: ROC analysis of level of HE4 measured before surgery and during the 3rd of chemotherapy to predict ovarian cancer relapse.

In *BRCA* mutated population, the ROC analysis has shown that a pre-surgery level of HE4 is a good prognostic marker to predict relapse of cancer (*p* = 0.019), and also the level of HE4 measured during the 3rd cycle of chemotherapy has given statistically significant results (Figure 2).

Sensitivity, specificity, area under the curve (AUC), likelihood ratio+, likelihood ratio− and post-test probability have been assessed (Table 5).

**Figure 3:**
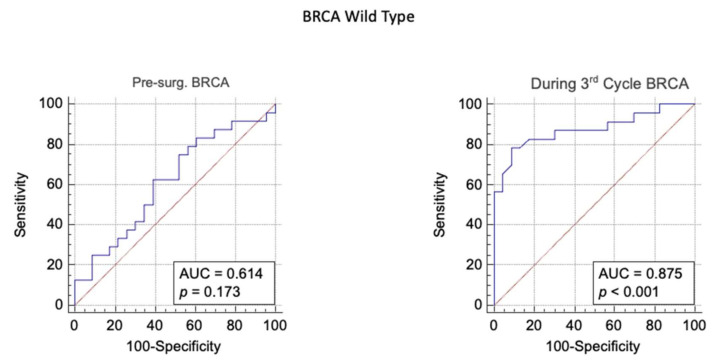
*BRCA* WT group: ROC analysis of level of HE4 measured before surgery and at the third cycle of chemotherapy to predict ovarian cancer relapse.

In wild-type *BRCA* patients the ROC curve analysis performed using the HE4 levels before the surgery has shown a non-statistically-significant result (*p* > 0.05). Whereas, the level of HE4 higher than 66 pmol/L after the third cycle of chemotherapy seems to be a good prognostic marker to identify those patients who will develop a relapse of the ovarian cancer (sensitivity = 78.26; specificity = 91.3; AUC = 87%, *p* value < 0.001; LR+ = 8.99; Post-test probability = 85%) (Figure 3).

The t-student test with Welch correction has shown that there is a statistically significant difference in HE4 levels between patients with a *BRCA* mutation and patients without a *BRCA* mutation (*p* value < 0.05). The pre-surgery levels in patients without a *BRCA* mutation has been a higher variability (*BRCA* + mean = 202.4; Standard deviation = 49.06 vs. *BRCA* – mean = 476.1 Standard deviation = 113.2). The *t*-test for the values after the 4th has not been shown for lack of data. 

## 4. Discussion

Despite several advances in cytoreductive surgery and the use of first-line chemotherapy, currently consisting in platinum-based protocols, followed by anti-angiogenetic monoclonal antibodies or Poly Adp Ribose Polymerase (PARP) inhibitors, as maintenance therapy, about 70% of patients affected by HSGOC, which initially responded to chemotherapy, could relapse [15,31].

Many studies are seeking a tool to early identify patients with platinum-resistant high serous epithelial ovarian cancer in order to improve and personalize the treatment worldwide.

In the last few years, a greater attention has been focused on the role of novel serum biomarkers in predicting chemosensitivity. Human Epididymis protein 4 (HE4) is one of the most promising markers [32], which demonstrated a good sensitivity and specificity in the diagnosis of ovarian cancer, overcoming the traditional role of Carbohydrate Antigen 125(CA125) [33].

HE4 also played an important role in detecting ovarian cancer recurrence with a better sensitivity than CA125 (91.3% vs. 52.2%, *p* value = 0.022).

Several papers also analyzed it as a prognostic factor; it has been measured at the time of diagnosis, before cytoreductive surgery, and subsequently during treatments and follow-up, and was compared with FIGO stage, residual tumor, and other well-known prognostic factors, proving to have a significant prognostic value itself [34,35,36].

Furthermore, both in vivo and in vitro studies analyzed the role of HE4 in predicting response to platinum chemotherapy, and all the studies agreed that HE4 could play as an earlier indicator of chemosensitivity or chemoresistance compared to CA125 [18,19].

Despite the small sample size, in the present pilot study, which certainly has limited statistical power, we found that, regardless of *BRCA* mutation, in both groups, a negative HE4 value at the third chemotherapy course seems to relate with good chemosensitivity and a higher probability of recurrence after 12 months (83%).

Similarly, a positive HE4 value at the third chemotherapy course seems to correspond to a good marker of chemoresistance with an even higher probability of recurrence within 12 months (96%).

This data was confirmed through the logistic regression analysis in which HE4 > 70 pmol/L after third cycle of CT is correlated with recurrence within 12 months (odds ratio = 28; 95% Cl = 4.55–183.18; *p* value < 0.0001).

The division of the whole sample allowed us to evaluate the differences between the two groups.

The median age of diagnosis is higher in *BRCA* WT group than in *BRCA* mutated group 64 (36–70) years vs. 51 (43–70) years, confirming the literature data that, in *BRCA* women, ovarian cancer usually manifests at an earlier age than in sporadic cases [1].

Even if having *BRCA* mutation is a well-demonstrated risk factor, among the individuals with OC, both *BRCA1* and *BRCA2*-associated patients have better outcomes.

Looking at the *BRCA* mutated group, we corroborated the literature data about the better prognosis of *BRCA* mutated patients [6].

In our sample, although small, the *BRCA* mutated group has a recurrence after 12 months in a greater proportion compared to the *BRCA* WT group (82% vs. 43%).

Another difference we found, through the t-student test with Welch correction, is about the preoperative HE4 value. It is higher in the *BRCA* WT group than in *BRCA* mutated group (843.2 (81.3–12,630) pmol/L vs. 293.8 (71–1498) pmol/L).

A possible reason for this difference in HE4 value could be that *BRCA* WT patients present major levels of microscopic disease. This possible explanation finds its basis in literature data in which HE4 value was largely related to tumor load. This explains the worse prognosis in patients *BRCA* WT, even though residual tumor after surgery is classified < 1 in both groups. 

The potential role of HE4 in predicting chemoresistant patients could be confirmed by the analysis of the two groups separately.

In the *BRCA* WT group, a negative HE4 value, at the III chemotherapy course matches good chemosensitivity with a higher probability of recurrence after 12 months (76%).

Similarly, a positive HE4 value at the III chemotherapy course corresponds to a good chemoresistant marker with a higher probability of recurrence within 12 months (96%). 

The statistical analysis suggests that the best balance between sensitivity and specificity seems to be the level of HE4 at the 3rd cycle. 

It should show that, in the *BRCA* population, a level of HE4 higher than 65.6 pmol/L after the 3rd cycle of chemotherapy seems to be a good prognostic marker to identify patient who will develop a relapse of ovarian cancer (sensitivity = 75; specificity = 99; AUC = 92%, *p* value < 0.001; LR+ = 750; Post-test probability = 99%).

Similar data were obtained in the analysis of the best balance between sensitivity and specificity is the level of HE4 at the 3rd cycle in the *BRCA* wild-type population.

In the *BRCA* mutated group, the role of HE4 seems to go even better. 

All the *BRCA* mutated patients with a negative HE4 value at the third cycle of chemotherapy were platinum-sensitive, while all the *BRCA* mutated patients with a positive HE4 value at the third cycle of CT were platinum-resistant.

The role of HE4 measurements during the 3rd cycle of chemotherapy in predicting chemo-recurrence was confirmed by the ROC analysis that has shown a significant trend.

Actually, it seems that the HE4 level could significantly classify the *BRCA* population into two different groups, the *BRCA* platinum-sensitive, and the *BRCA* platinum-resistant patients.

According to this, HE4, combined with mutational BRCA screening test, could become part of a decisional algorithm, as our group did with the REM algorithm for endometrial cancer [37], in order to decide the proper therapy for each woman affected by ovarian cancer.

The importance to detect platinum-resistant patients in advance, before tumor relapse, opens the door to new scenarios.

First of all, the opportunity to select a high-risk relapse group allows us to avoid unnecessary chemotherapy courses, reducing toxicity related to chemotherapy, which typically resulted in neutropenia and nephrotoxicity.

Moreover, literature data demonstrated that Promoter CGI methylation revealed a positive association between the total number of hypermethylated CGIs and GI50 values (i.e., increased drug resistance) following successive cisplatin treatment cycles [38]. Hypothetically, this algorithm may predict the individual response to platinum-based chemotherapy and may eventually allow us to change the chemotherapy regimen before the end of the six standard cycles of chemotherapy in patients identified as “platinum non responders”.

The platinum-resistant *BRCA* mutated patients are about 10% of all *BRCA* patients, and it might be crucial to detect them because they have a better response to new chemotherapy drugs, such as PARP-inhibitors.

The possibility to shift earlier to another chemotherapy treatment, once proved that the first-line was ineffective, allows us to have many positive effects, also in terms of economic benefits.

## 5. Conclusions

In conclusion, we argue that a positive value of HE4 at the III chemotherapy course could represent a predictive factor for chemoresistance in both *BRCA* carriers. Moreover, *BRCA* mutated patients do not have better chemosensitivity than wild-type ones, and the HE4 predicting role of chemoresistance is not higher in *BRCA* population.

The main weakness of our study is the small set of patients and also the discrepancy between the number of *BRCA* wild-type patients and *BRCA* mutated ones. We strongly believe that HE4 performance should be tested in the follow-up of a larger population of EOC patients in prospective, multicentric, and randomized trials, in order to improve the surveillance strategies and treatment options. Therefore, it would be interesting to investigate the relationship between HE4 levels and somatic and germline *BRCA* mutations in predicting platinum response, in a multivariate analysis.

The challenge to predict platinum-resistant and to anticipate second-line treatments, improving quality of life and overall survival, is still entirely open.

## Figures and Tables

**Table 1:** Patient demographics and postoperative data.

Variables	BRCA WT (53 pts)	BRCA MUT (16 pts)
AGE (years) Mean (range)	64 (36–70)	51 (43–70)
PERFORMANCE STATUS (*ECOG 0*–*5)*		
- 0	45 (85%)	13 (81%)
- 1	8 (15%)	3 (19%)
FIGO stage n (%)		
- I	7 (13%)	2 (12.5%)
- II	9 (17%)	2 (12.5%)
- III	34 (64%)	9 (56%)
- IV	3 (6%)	3 (19%)
RESIDUAL TUMOR *n* (%)		
- 0	32 (60%)	10 (63%)
- < 1	21 (40%)	6 (37%)
HISTOLOGICAL SUBTYPE		
High-Grade Serous Ovarian Cancer *n* (%)	53 (100%)	16 (100%)
PLATINUM-SENSITIVE *n* (%)	23 (43%)	13 (81%)
PLATINUM-RESISTANCE *n* (%)	30 (57%)	3 (19%)

**Table 2:** All population: Correlation between HE4 value and recurrence.

All Population		Platinum-Sensitive	Platinum-Resistant	Total
HE4 value at the III cycle of CT *	HE4 negative < 70 pmol/L	35 (83%)	7(17%)	42 (61%)
	HE4 positive > 70 pmol/L	1 (4%)	26 (96%)	27 (39%)

* chemotherapy

**Table 3:** *BRCA* WT group: Correlation between HE4 value and recurrence.

*BRCA* WT		Platinum-Sensitive	Platinum-Resistant	Total
HE4 value at the III cycle of CT	HE4 negative < 70 pmol/L	22 (76%)	7 (24%)	29 (100%)
	HE4 positive > 70 pmol/L	1 (4%)	23 (96%)	24 (100%)

**Table 4:** *BRCA* mutated group: Correlation between HE4 value and recurrence.

*BRCA* MUTATED		Platinum-Sensitive	Platinum-Resistant	Total
HE4 value at the 3rd cycle of CT	HE4 negative < 70 pmol/L	13 (100%)	0	13 (100%)
	HE4 positive > 70 pmol/L	0	3 (100%)	3 (100%)

**Table 5:** The area under the curve, cut-off value, sensitivity, specificity, *p* value, likelihood ratio+, likelihood ratio− and post-test probability resulting from ROC analysis of level of HE4 measured before surgery, during the third chemotherapy to predict ovarian cancer relapse.

	Pre Test Probability	Area Under the Curve	Younden Index	Sensitivity	Specificity	*p* Value	Likehood Ratio+	Likehood Ratio−	Post Test Odd	Post Test Probability
During 3rd Cycle *BRCA*	0.4	0.92	> 66	75	99.9	*p* < 0.001	750	0.25	500	0.99
During 3rd Cycle WT	0.4	0.87	> 66	78	91	*p* < 0.001	8.9	0.24	5.9	0.86
Pre-Surg. *BRCA*	0.4	0.79	> 1767	99,9	73	*p* = 0.019	3.7	0.001	2.4	0.71
Pre-Surg. WT	0.4	0.61	> 231	63	61	*p* = 0.17	1.6	0.62	1.06	0.52

## Data Availability

The data presented in this study are available on request from the corresponding author. The data are not publicly available due to patient’s privacy policy.
